# Effect of vertically aligned carbon nanotube density on the water flux and salt rejection in desalination membranes

**DOI:** 10.1186/s40064-016-2783-3

**Published:** 2016-07-22

**Authors:** Samarth Trivedi, Kamal Alameh

**Affiliations:** Electron Science Research Institute, Edith Cowan University, Joondalup, WA 6027 Australia

**Keywords:** Water desalination, Nano-membranes, Vertically aligned carbon nanotubes

## Abstract

In this paper, vertically aligned carbon nanotube (VACNT) membranes of different densities are developed and their performances are investigated. VACNT arrays of densities 5 × 10^9^, 10^10^, 5 × 10^10^ and 10^11^ tubes cm^−2^, are initially grown on 1 cm × 1 cm silicon substrates using chemical vapour deposition. A VACNT membrane is realised by attaching a 300 μm-thick 1 cm × 1 cm VACNT array on silicon to a 4″ glass substrate, applying polydimethylsiloxane (PDMS) through spin coating to fill the gaps between the VACNTs, and using a microtome to slice the VACNT–PDMS composite into 25-μm-thick membranes. Experimental results show that the permeability of the developed VACNT membranes increases with the density of the VACNTs, while the salt rejection is almost independent of the VACNT density. The best measured permeance is attained with a VACNT membrane having a CNT density of 10^11^ tubes cm^−2^ is 1203 LMH at 1 bar.

## Background

Within the last one and half decades, many researchers have worked on different types of CNT based membranes. Vertically aligned carbon nanotubes (VACNT) embedded in a polymer matrix have been developed and tested for gas and liquid transport and filtration. Hinds et al. (2004) have pioneered the multiwall carbon nanotubes (MWCNTs) sealed membrane and observed that liquid transportation was much faster than that predicted by the hydrodynamic theory. Holt et al. (2006) have adopted the same concept and developed a membrane using a chemical vapour deposited (CVD) double-wall carbon nanotube (DWCNT) matrix in silicon nitride. Gas transportation was more than one order rapid than predicted by the Knudsen diffusion model. Kim et al. (2007) have used single-wall carbon nanotubes and incorporated them into existing membranes. The space between CNTs was filled with polymer and the permeance of the membrane for various gases was investigated, demonstrating a reduction in permeability, mainly caused by the polymer layer.

In all above-mentioned works, the total flux (of liquid or gas) was typically dependent on the type of the used CNTs and their densities. As each membrane structure was prepared for a specific application, with different polymer materials being used to fill the space between CNTs, no definite conclusion has so far confirmed the effect of CNT density on the membrane’s performance. Conventionally, VACNT membranes have been fabricated using either compression and rolling techniques (Yu et al. 2009), with the main aim of research being to improve the membrane permeability without affecting the salt rejection property.

Recently, Wang et al. have reported wafer-scale transfer of VACNT arrays (Wang et al. 2014), demonstrating that after a short time of weak oxidation, VACNTs can be easily detached from the native growth substrates, and thus, a freestanding VACNT film can be obtained. This demonstration opens the way for the development of large-size VACNT-based membranes by transferring multiple VACNT films onto large-scale membranes (or substrates) for commercial applications.

In this paper, the permeance and salt rejection properties of four membranes of different VACNT densities are experimentally investigated. The developed VACNT membranes display adequate permeability and salt rejection in comparison with previously reported membranes (Hinds et al. 2004; Holt et al. 2006; Kim et al. 2006, 2007; Yu et al. 2009; Sharma et al. 2010).

## Experimental method

**Fig. 1:**
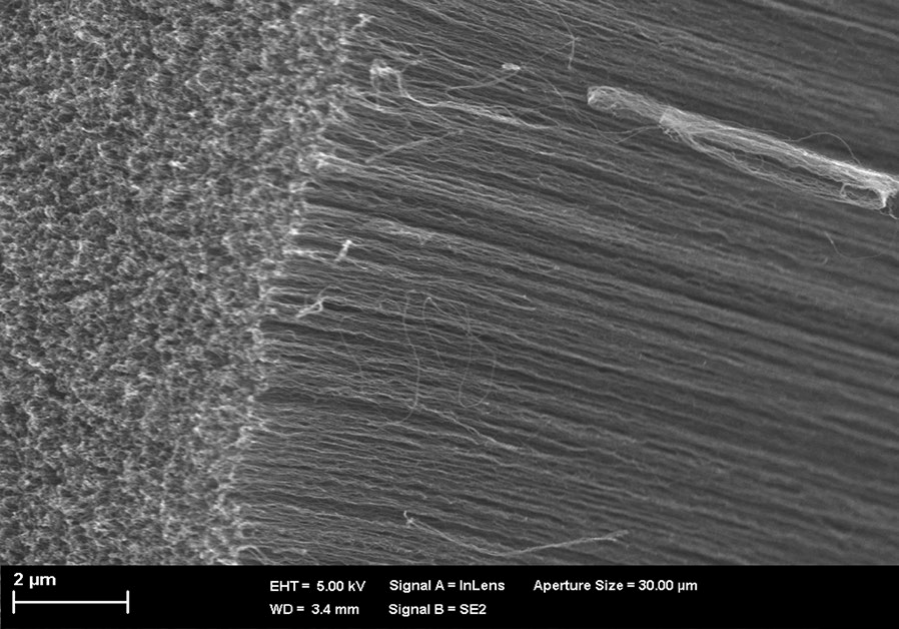
Cross-section of a developed VACNT array, captured using an FIB-SEM (Focussed ion beam scanning electron microscope, Zeiss—Neon 40 EsB)

The CNTs, grown on Si wafer, of different densities were purchased from DK Nanomaterials Co. Ltd (China). The average outer diameter of the VACNTs was 8 nm and their length was around 300 μm. Figure 1 shows a cross-section of one of the developed VACNTs, captured using an FIB-SEM (Focussed ion beam scanning electron microscope, Zeiss—Neon 40 EsB). The SEM image of the VACNT wafer shown in Fig. 1 was obtained by simply placing it on a stage of variable tilting angle and using a ZEISS-NEON 40ESP FIB/SEM system. The silicon wafer onto which the VACANTs were grown was glued onto a glass surface and placed in a spin coater, where 50 % (W/w) poly(dimethylsiloxane) (PDMS) in xylene was added drop by drop at a spin speed of 2500 rpm. It is important to note that the Si substrate was specifically used for the growth of VACNTs in a chemical vapour deposition (CVD) system. Throughout the experiments, the Si substrates of the various VACNT wafers were glued onto glass substrates, which were retained as mechanical supports only. Hence, the Si substrates did not contribute to any chemical reaction. After spin coating the PDMS onto the VACNT wafer, VACNT membranes of different thicknesses were sliced and detached from the Si/glass supports.

**Fig. 2:**
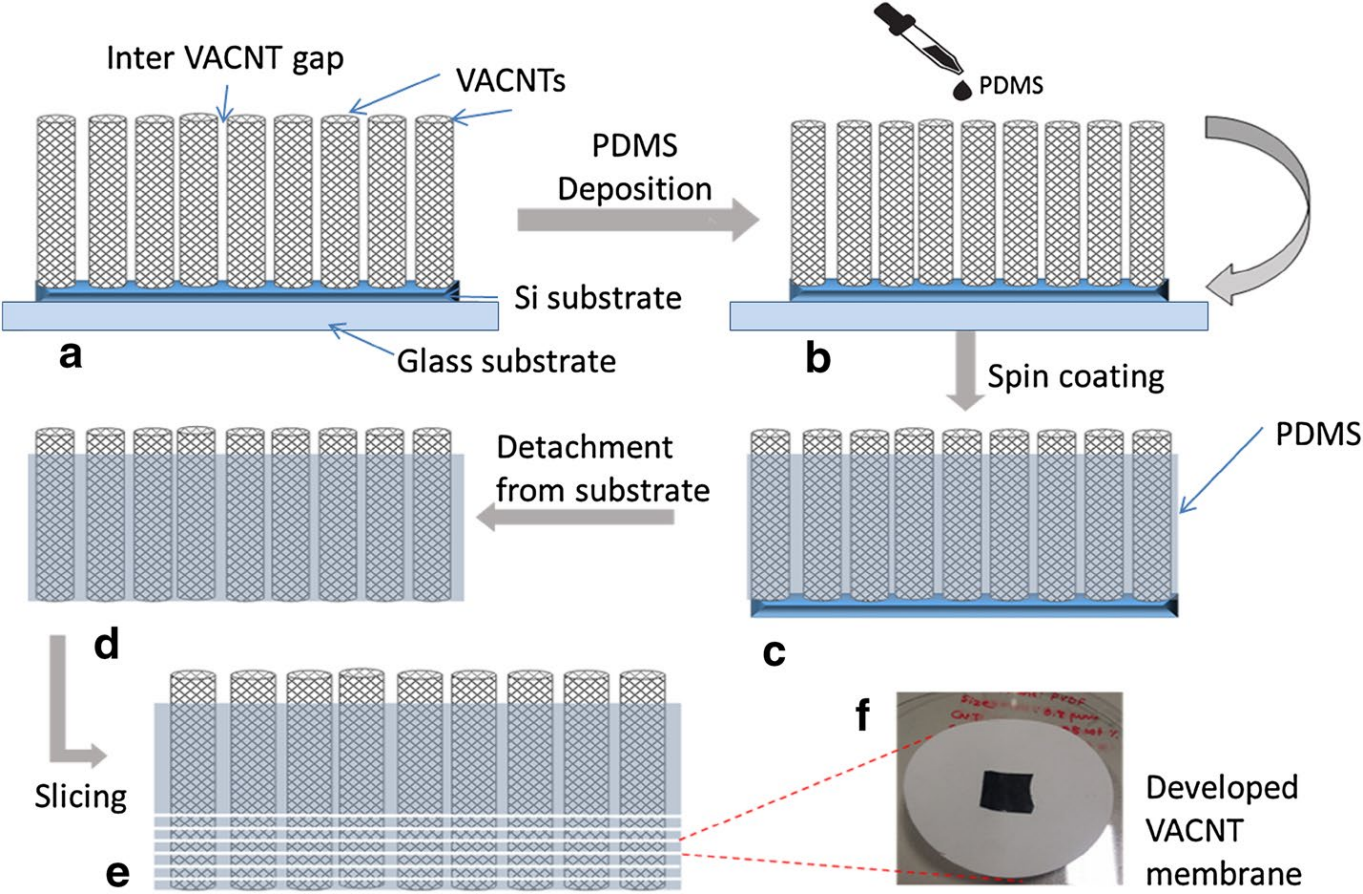
VACNT membrane fabrication process. **a** VACNT on silicon substrate glued to glass. **b** PDMS is added. **c** PDMS is cured through heating in a vacuum oven, and the volatile part of the PDMS is evaporated. **d** Silicon and glass substrates are removed using mechanical peeling. **e** VACNT–PDMS composite is sliced using a microtome machine. **f** Photograph of one of the developed VACNT membranes

The sample was then dried in a vacuum oven at 100 °C for 6 h. During this time, the volatile portion of the PDMS material evaporated, resulting in cured PDMS between the VACNT. The VACNT–PDMS composite was then detached from the silicon substrate through mechanical peeling before slicing it into 25 µm-membranes, using a microtome machine. The membranes were then placed onto a polyvinylidene fluoride (PVDF) support layer (Du et al. 2011; Srivastava et al. 2004). The average pore size of the PVDF support layer was 200 nm. The complete development process is illustrated graphically in Fig. 2.

Drops of liquid PDMS were added to and spread over the VACNTs using a spin coater operating at 2500 rpm. It is typically possible that some PDMS enters into the CNTs from the opening area, however, due to its high dynamic viscosity 3500 Centipoise (obtained from product data sheet of Sylgard 184, Dow Corning), the PDMS does not enter deeply into the CNTs. Thus, by slicing the VACNT–PDMS composite block into 25 µm thick slices and discarding the top slice, CNT blockage by PDMS is minimised.

PDMS was diluted using xylene and a sample was placed in a vacuum desiccator to remove any air trapped by the PDMS. SEM images were taken after every fabrication step and SEM images of the final samples are reported in the manuscript. The various VACNT membranes were purchased from DK Nanomaterials Co. Ltd. Company, which also measured the dimensions of CNTs using TEM and their densities using SEM.

The fabrication method is summarised as follows: VACNTs on silicon wafers of different VACNT densities were purchased from DK Nanomaterials Co. Ltd (China). A two-step fabrication process was used to develop the membranes. First, PDMS was deposited onto the purchased VACNTs using spin coating; second, 25 µm thick membranes were sliced out of the VACNTs + PDMS block using a microtome machine. Figure 2 shows the fabrication steps used for the development of the VACNT membranes. A glass substrate was uses as a mechanical support onto which the Si wafer (which has the VACNTs) was glued. The glass support was subsequently removed before the VACNT membranes were sliced.

**Fig. 3:**
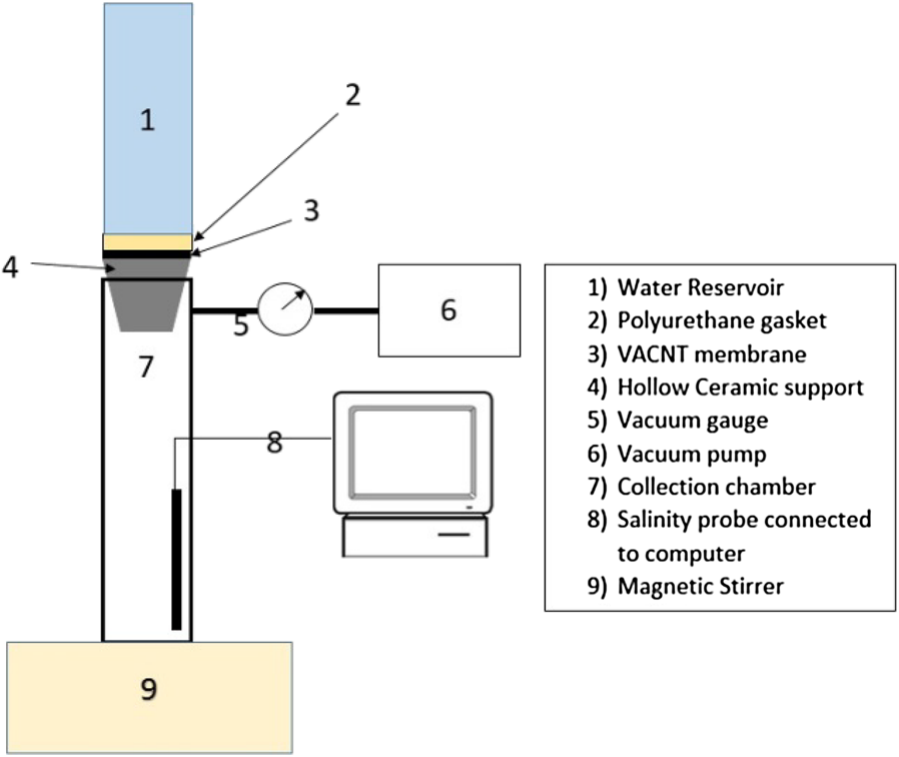
Modified dead-end filtration setup used to investigate the performances of the developed VACNT membranes

Experiments were performed using the dead-end filtration setup shown in Fig. 3, where feed flow through the membrane is forced using a vacuum pump rather than direct pressure (Srivastava et al. 2004). The dead end cell comprised a bottom collection chamber with a magnetic stirrer, ceramic support onto which the VACNT membrane was placed, a polyurethane gasket (sealer) that prevented water/gas leakage through the membrane edges and a water reservoir. The VACNT membrane was fed from a water reservoir containing water of salinity initial 10,000 ppm, and a vacuum pump was used to create pressure gradient that enables water to flow through the membrane. During the experiments, negative pressure was applied to a modified dead-end cell setup, with ambient pressure (780 torr) at the feed side and vacuum of 640 torr applied at collection side. The vacuum pressure at the permeate side was monitored by a pressure gauge and the quality of permeate was monitored using a salinity sensor. As vacuum was applied to the container collecting permeate, the solution was automatically degassed, and hence, a degassed solution was used to measure the water flux. The volume of the collected permeate was recorded every minute for 60 min. Note that, as illustrated in Fig. 3, a decrease in flux with time was experienced since the feed was not stirred as permeate.

Table 1 shows the parameters used to calculate permeability and the enhancement factor for each membrane.

**Table 1:** Vertically aligned carbon nanotubes (VACNT) membrane parameters used to calculate the permeability and enhancement factor

VACNTs density (tubes cm^−2^)	CNT diameter (nm)	Dynamic viscosity µ (Pa S) at 20 °C	Pressure difference Δp (torr)	Membrane thickness (µm)
5 × 10^9^–1 × 10^11^	8	1.002 × 10^−3^	140	25

The permeate flux for each membrane was measured under different vacuum pressures in order to check the consistency and reproducible fabrication of the membranes. The standard conditions for the evaluation of membranes were 20° C and 2 bar. The pure water flux was calculated using the following equation (Du et al. 2011; Srivastava et al. 2004; Anh et al. 2012; Zhang et al. 2014; Cooper et al. 2003; Youngbin et al. 2014; Vatanpour et al. 2011)

1 $$Q = \frac{M}{{A\Delta t}}$$

where M is the weight of permeate water (kg), A is the membrane area (m^2^), Δt is the permeation time (h).

The salt rejection was calculated from the measured flux, for all developed membrane samples using the following equation (Vatanpour et al. 2011)

2 $$R \left( \% \right) = \left. {\left( {1 - \frac{{C_{p} }}{{C_{f} }} } \right.} \right)^{{}} \times 100$$

where R is rejection, C_p_ is concentration of permeate and C_f_ is concentration of feed.

## Result and discussion

**Fig. 4:**
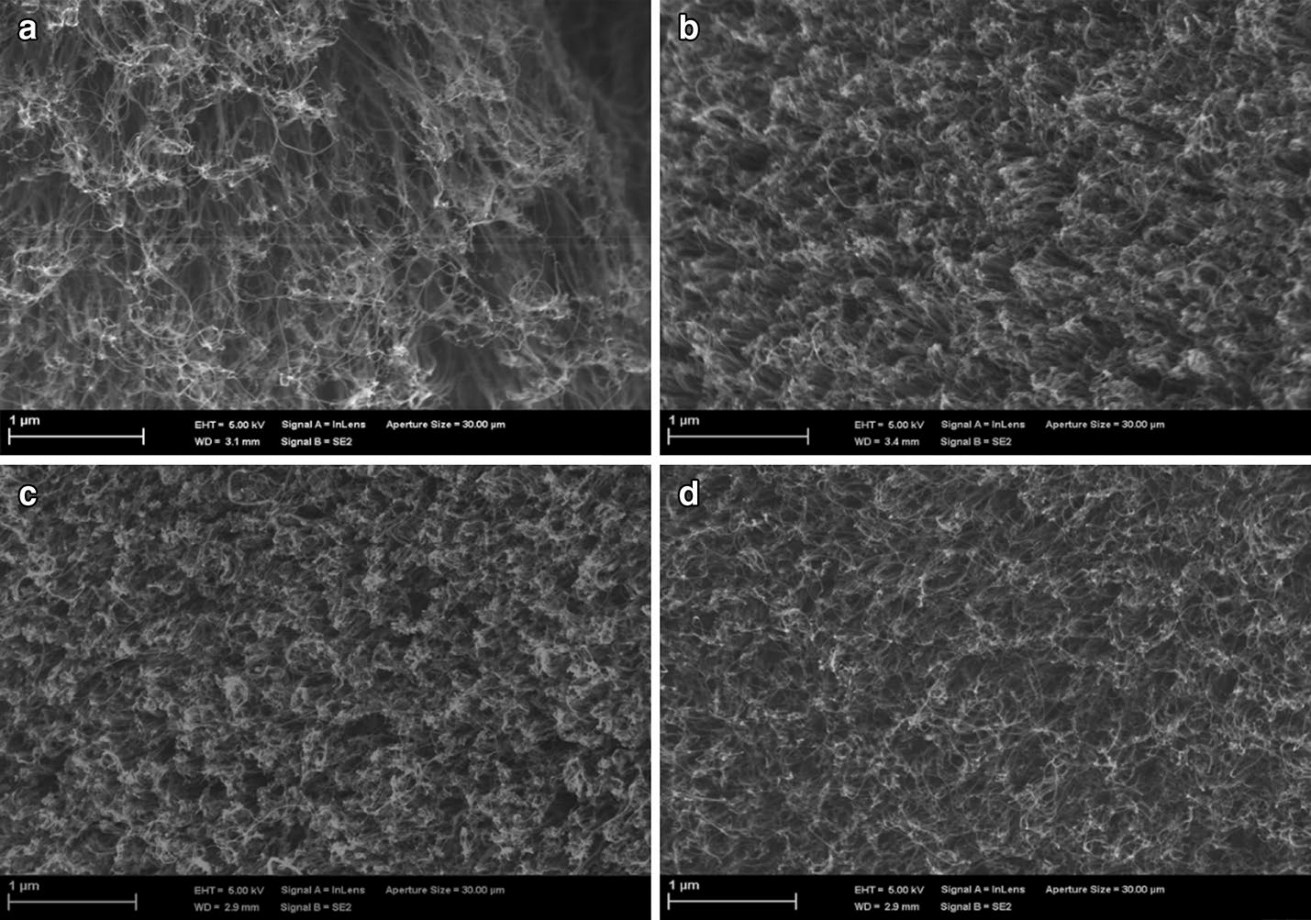
SEM images of the surfaces of the four VACNT membranes of densities, **a** 5 × 10^9^, **b** 10^10^, **c** 5 × 10^10^ and **d** 10^11^ tubes cm^−2^ before slicing

**Fig. 5:**
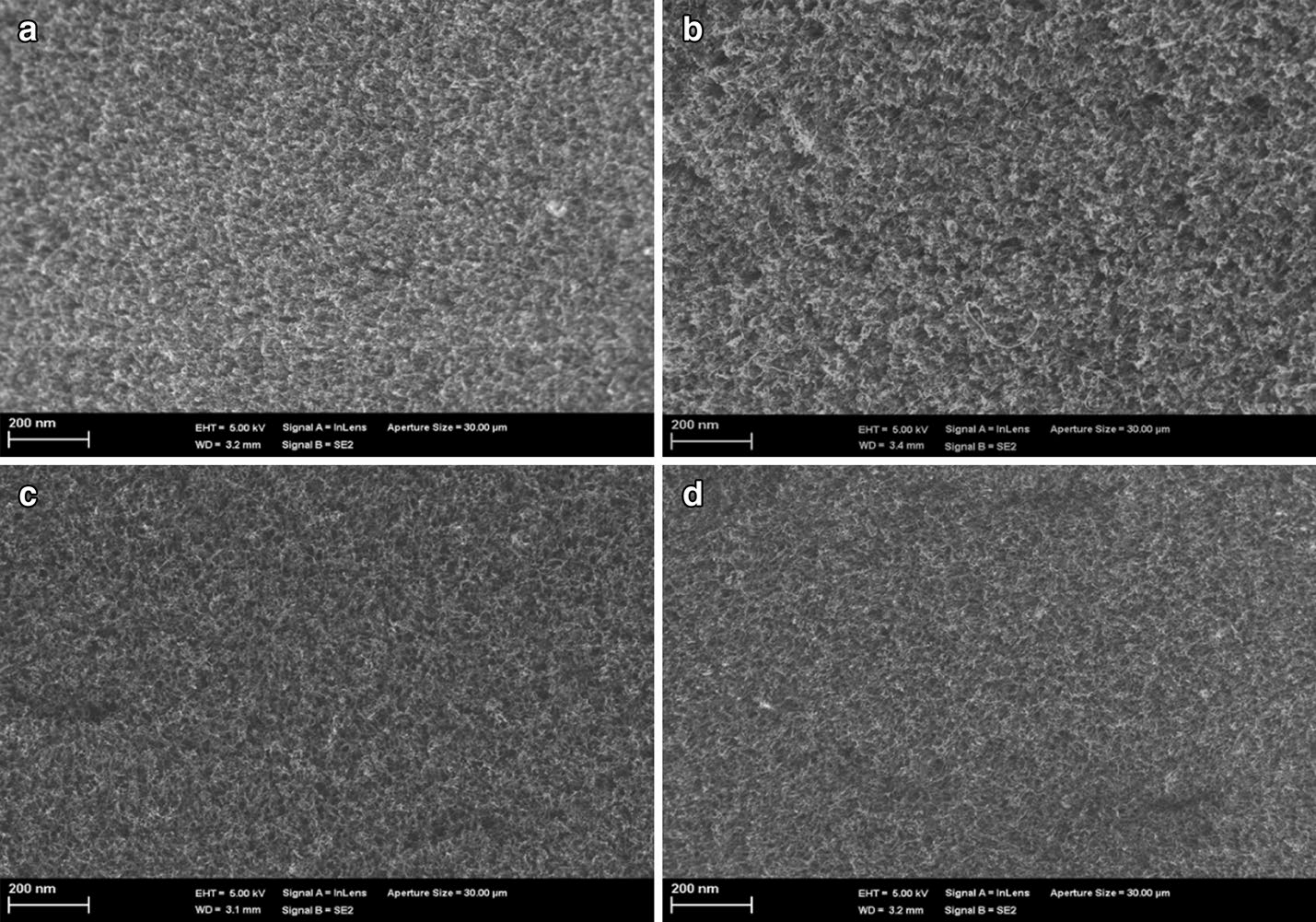
SEM images of the surfaces of the sliced 25 μm-thick membranes of densities, **a** 5 × 10^9^, **b** 10^10^, **c** 5 × 10^10^ and **d** 10^11^ tubes cm^−2^

Figure 4a–d show SEM images of the surfaces of the four VACNT membranes of densities, 5 × 10^9^, 10^10^, 5 × 10^10^ and 10^11^ tubes cm^−2^, respectively, before slicing. It is obvious from Fig. 4 that, before slicing, the VACNT–PDMS composites were slightly protruded from the surface. The cracks and void spaces between the VACNT were checked to ensure that all surfaces of the membranes were fully filled with PDMS. Figure 5a–d show SEM images of the surfaces of the sliced 25 µm-thick membranes after slicing. Close examination of the membrane’s surfaces shows that some of the VACNTs were entangled or tilted due to the sheer forces of the polymer droplets or the centrifugal forces during spin coating. However, the affected areas were typically negligible, compared to the total area of the individual membranes.

**Fig. 6:**
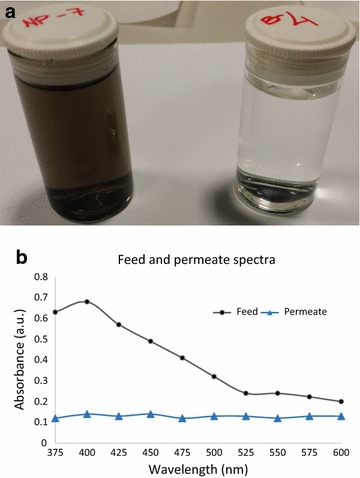
**a** Typical images of iron oxide nanoparticle solution and liquid filtered by one of the developed VACNT membranes. **b** UV–Vis spectra of the iron oxide solution at the feed side and the collected permeate

Experiments were carried out to test the ability of the developed VACNT membranes to filter water-soluble iron oxide (Fe_2_O_3_) nanoparticles present in the DI water, whose average diameter was 10 nm (Zhang et al. 2015). A solution of iron oxide was added to the above-described modified dead end setup at a pressure of 2 bar and permeate was collected. Figure 6 shows visual difference in the solution of iron oxide and filtered water. Both liquids are tested using a UV–visible spectrometer. Figure 6 demonstrates the ability of VACNT membranes to produce clear and colourless permeate water. The 404 nm surface plasmon resonance band of the iron oxide nanoparticles is visible in the feed solution. However, the collected permeate shows no sign of the presence of nanoparticles. The solution of iron oxide was used only to show “size exclusion” not to investigate the “salt rejection” capability of the developed VACNT membranes.

The results shown in Fig. 6 also indicate that the average diameter of the CNT is less than 10 nm (average iron oxide nanoparticle size) and that the gaps between the VACNT were completely occupied by PDMS (Vatanpour et al. 2011; Zhang et al. 2015; Zhao et al. 2009a).

The properties of the fabricated VACNT membranes were compared with the properties of CNT membranes developed by other groups, which were mainly used for gas filtration (Futaba et al. 2006; Zhao et al. 2009b; Ge et al. 2012; Skoulidas et al. 2002; Ackerman et al. 2003; Kumar et al. 2012; Gilani et al. 2013; Majumder et al. 2011; Krishnakumar et al. 2012; Mi et al. 2007). Table 2 lists the types and characteristics of the reported CNT membranes. Note that this table only provides useful information, rather than a comparison analysis, on reported VACNT membranes, since theses membranes are not structurally similar and were used for different applications. As shown in Table 2, most reported VACNT densities were between 10^9^ to 2.5 × 10^11^ tubes cm^−2^ and all types of CNTs were used, namely, single-walled (SWNTs), double-walled (DWNTs) or multi-walled (MWNTs). It is important to note that various VACNT membranes of thicknesses 22, 25 and 30 μm were developed, and results show that the impact of the VACNT membrane thickness on the membrane’s performance (flux and salt rejection) is negligible.

**Table 2:** Parameters of key VACNT membranes developed by other groups (Futaba et al. 2006; Zhao et al. 2009b; Ge et al. 2012; Skoulidas et al. 2002; Ackerman et al. 2003; Kumar et al. 2012; Gilani et al. 2013; Majumder et al. 2011; Krishnakumar et al. 2012; Mi et al. 2007)

CNT membrane	Our group	Mi group (Mi et al. 2007)	Hinds group (Hinds et al. 2004)	Holt group (Holt et al. 2006)	Kim group (Kim et al. 2007)
Main structure	VACNT + PDMS composite	Porous aluminium support	Free standing	Silicon water	PTFE Filter
Filler material	PDMS	Polystyrene	Polystyrene	Silicon nitride	Polysulfone
CNTs	MWCNT	MWCNT	MWCNT	DWCNT	SWCNT
Average outer diameter (nm)	20	20	NA^a^	2	NA
Average inner diameter (nm)	8	6.3	7.5 ± 2.5	1.6 ± 0.4	1.2
Thickness of CNT layer (μm)	25	~10	5–10	5	6
CNT density (tubes cm^−2^)	5 × 10^9^, 10^10^, 5 × 10^10^, 10^11^	1.87 × 10^9^	6 × 10^10^	2.5 × 10^11^	(7.0 ± 1.75) × 10^10^
Maximum permeance (LMHBar)	917, 1007, 1111, 1203	475	1100	1080	NA

Note that theses membranes are not structurally similar and were used for different applications

*SWNT* single-walled carbon nanotube, *DWCNT* double-walled carbon nanotube, *MWCNT* multi-walled carbon nanotube

^a^Not available data

It is obvious from Table 2 that the maximum fluxes (rounded to nearest integer) are 917, 1007, 1111 and 1203 LMH for the VACNT densities of 5 × 10^9^, 1 × 10^10^, 5 × 10^10^ and 1 × 10^11^ tubes cm^−2^, respectively. Note that in order to confirm the accuracy of our experimental results, the performance of the VACNT membrane were compared with that reported by Hinds et al., which has a CNT density (6 × 10^10^ tubes cm^−2^) that is slightly less than that of the third membrane developed in this work (of density 5 × 10^10^ tubes cm^−2^). Table 2 shows that the water flux achieved using our membrane is slightly higher than that achieved by Hinds et al., who used Polystyrene as the filler material.

Table 3 shows the salt rejection properties of key reported VACNT membranes. It is obvious from Table 3 that the proposed VACNT membranes exhibit high salt rejection in comparison with reported CNT-based membranes.

**Table 3:** Salt rejection performance achieved by key reported CNT membrane types

CNT density	CNT inner diameter (nm)	Membrane type	Salt rejection (%)
2.5 × 10^11^ (Corry 2008)	0.8	Vertically aligned (VA)	100
2.5 × 10^11^ (Corry 2008)	1.5	Vertically aligned (VA)	95
5 × 10^9^ (this paper)			96.92
1 × 10^10^ (this paper)	5	Vertically aligned	96.99
5 × 10^10^ (this paper)		(VA)	97.10
1 × 10^11^ (this paper)			97.26
20 wt% CNT (Thomas and Corry 2015)	1.5	Mixed matrix (MM)	93
0.05 wt% CNT (Ocvirk et al. 2000)	5	Mixed matrix (MM)	87

**Fig. 7:**
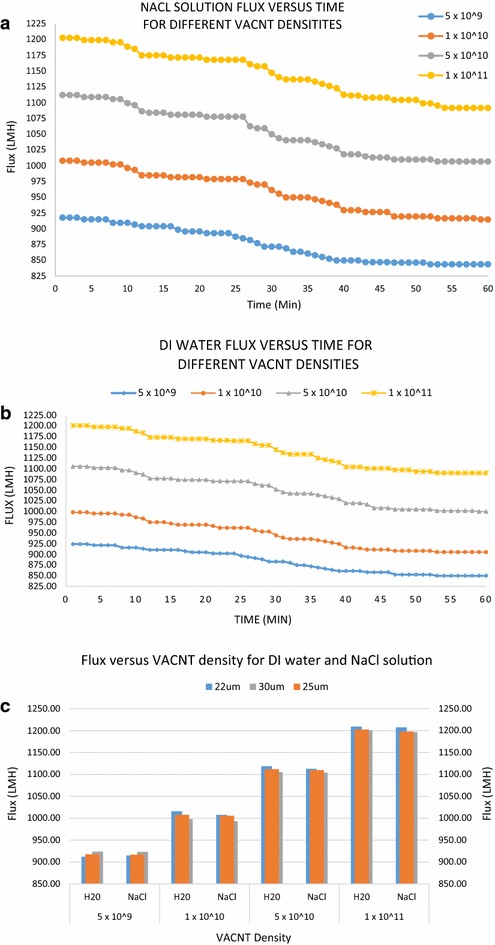
**a** NaCl solution flux (LMH) versus time for the different developed VACNT membranes, over a period of 60 min. **b** DI water flux versus time for different VACNT densities. **c** Flux versus VACNT density for DI water and NaCl solution, for different VACNT membrane thicknesses

Figure 7a, b show the flux versus time for different VACNT densities, for NaCl solutions and DI water, respectively. The flux was measured by using DI water feed and NaCl solutions containing 10,000 ppm of NaCl, and monitoring the total amount of water permeate collected after filtration by the developed VACNT membranes. The fluxes for pure DI water as well as 10,000 ppm NaCl solutions were measured, and found to be almost similar, as evident from Fig. 7a, b. Therefore, for solutions containing less than 10,000 ppm NaCl, the salt content has negligible impact on the flux.

It is important to notice from Fig. 7a, b that, for both NaCl solutions and DI water, the increase in flux is not directly proportional to the VACNT density. This is because when the density of VACNT increases, the number of CNT walls also increases, while the active inner diameter of CNT remains the same. Therefore, the slight increase in flow rate is attributed to additional small volumes of water flowing between walls of the MWCNTs. Note also that, the main advantage of increasing the VACNT density is the prevention of membrane biofouling, while achieving a slight increase in flow rate, with negligible impact on the salt rejection (Youngbin et al. 2014). Figure 7c compares the flux attained with DI water and NaCl solutions for different VACNT densities.

Note that the charge-based filtering mechanism, exhibited in the proposed VACNT filters, allows a relatively high CNT diameter to achieve better salt rejection than size-based filtering counterparts. This is due to the electrical and surface properties (Zeta potential and surface roughness, respectively) of PDMS, which are the key factors affecting ion transportation through CNTs (Schrott et al. 2009). Note also that the energy barrier of the CNT pores for Na+ ions depends on the pressure, temperature and concentration of the ions in the feed (Schrott et al. 2009; Corry 2008).

**Fig. 8:**
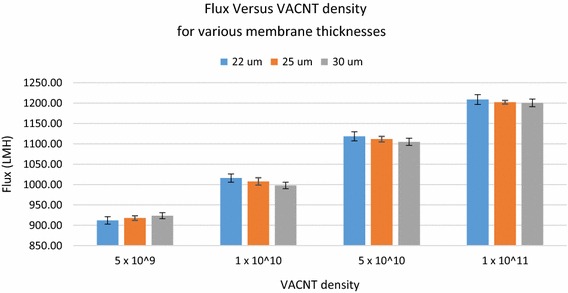
Flux versus VACNT density for different membrane thicknesses. *Error bars* show the maximum deviation in flux for the various membrane thicknesses

Figure 8 shows the flux versus VACNT density for different membrane thicknesses. Error bars show the maximum deviation in flux for the various membrane thicknesses that were tested. It is obvious from Fig. 8 that a small variation in the VACNT membrane thickness (±5 µm around 25 µm) has a negligible impact on the flux.

**Fig. 9:**
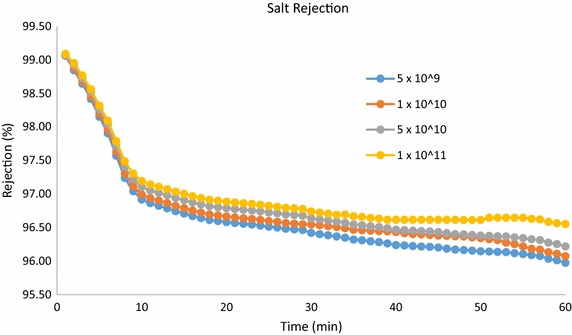
Salt rejection versus time, for the different developed VACNT membranes

Figure 9 shows the salt rejection versus time for the various developed VACNT membranes. This was carried out by measuring the salinity of the collected water permeate at time intervals of 1 min, using a Vernier salinity probe (Majumder et al. 2005; Sears et al. 2010; Verweij et al. 2007). A conductivity probe was used to measure the salt concentration of the collected permeate, and based on this measurement the salt rejection was simply calculated using Eq. (2). This is the simplest approach to accurately measuring the salt rejection.

The salt ion rejection depends on two main factors, (1) inner diameter of the carbon nanotubes (the average inner diameter of MWCNT is 5 nm) and (2) the surface charge of the material used to fabricate the membrane. Salt rejection reduces with increasing the diameter of the CNTs (Thomas and Corry 2015). A native PDMS surface is typically negatively charged as demonstrated by Ocvirk et al. (2000). Therefore, the Na+ ions are trapped by the PDMS surface, hence increasing the salt rejection of the PDMS–CNT membrane. During the experiments, initially, the surface charge of the membrane was high, since both the low CNT diameter and high surface charge of the membrane contributed to the salt rejection. After 60 min of filtration, salt ions accumulated on the surface of the membrane, thus reducing the salt rejection contributed by the surface charge of the membrane, as shown in Fig. 7, wherein the results are in agreement with the investigation reported by Schrott et al. (2009).

Note that, the concentration polarisation (due to the accumulation of rejected salt particles at the membrane surface) typically reduces the salt rejection capability of the VACNT membranes and negatively influences mass transfer, thus increasing the osmotic pressure and reducing the water flux at the feed side. Concentration polarisation can be overcome by osmotic backwash, which is typically induced when the feed-side osmotic pressure exceeds the applied hydraulic pressure across the membrane (Chen et al. 2004; Juang et al. 2008).

It is obvious from Fig. 9 that all developed VACNT membranes displayed similar salt rejection properties. The experimental results shown in Figs. 7 and 8 demonstrate the ability of the developed VACNT membranes to achieve RO filtration water and high fluxes, in addition to preventing biofouling (Youngbin et al. 2014). The ability of VACNT membranes to prevent biofouling has been reported by Youngbin et al. (2014). This manuscript mainly focuses on comparing the water flux and salt rejection VACNT-based membranes of different densities. A comparison between the biofouling properties of the various developed VACNT membranes will be addressed in detail along with different types of CNTs in future publications.

## Conclusion

The performance of VACNT membranes of densities 5 × 10^9^, 10^10^, 5 × 10^10^ and 10^11^ tubes cm^−2^ have been developed and their performances investigated. The VACNT membrane development process has been described in detail. Experimental results have confirmed that the permeability of VACNT membranes increases with the density of the VACNT, while the salt rejection is almost independent of the VACNT density. A permeance of 1203 LMHBar and salt rejection exceeding 96.5 % have been experimentally achieved using a VACNT membrane of VACNT density around 10^11^ tubes cm^−2^.
